# Saturation Diving Alters Folate Status and Biomarkers of DNA Damage and Repair

**DOI:** 10.1371/journal.pone.0031058

**Published:** 2012-02-07

**Authors:** Sara R. Zwart, J. Milburn Jessup, Jiuping Ji, Scott M. Smith

**Affiliations:** 1 Universities Space Research Association, Houston, Texas, United States of America; 2 National Cancer Institute, Bethesda, Maryland, United States of America; 3 NASA Johnson Space Center, Houston, Texas, United States of America; Ospedale Pediatrico Bambino Gesu', Italy

## Abstract

Exposure to oxygen-rich environments can lead to oxidative damage, increased body iron stores, and changes in status of some vitamins, including folate. Assessing the type of oxidative damage in these environments and determining its relationships with changes in folate status are important for defining nutrient requirements and designing countermeasures to mitigate these effects. Responses of humans to oxidative stressors were examined in participants undergoing a saturation dive in an environment with increased partial pressure of oxygen, a NASA Extreme Environment Mission Operations mission. Six participants completed a 13-d saturation dive in a habitat 19 m below the ocean surface near Key Largo, FL. Fasting blood samples were collected before, twice during, and twice after the dive and analyzed for biochemical markers of iron status, oxidative damage, and vitamin status. Body iron stores and ferritin increased during the dive (*P*<0.001), with a concomitant decrease in RBC folate (*P*<0.001) and superoxide dismutase activity (*P*<0.001). Folate status was correlated with serum ferritin (Pearson *r* = −0.34, *P*<0.05). Peripheral blood mononuclear cell poly(ADP-ribose) increased during the dive and the increase was significant by the end of the dive (*P*<0.001); γ-H2AX did not change during the mission. Together, the data provide evidence that when body iron stores were elevated in a hyperoxic environment, a DNA damage repair response occurred in peripheral blood mononuclear cells, but double-stranded DNA damage did not. In addition, folate status decreases quickly in this environment, and this study provides evidence that folate requirements may be greater when body iron stores and DNA damage repair responses are elevated.

## Introduction

Saturation dives involve long-duration (several days to weeks) stays in a closed environment with an increased partial pressure of oxygen. Saturation diving and exposure to oxygen-rich environments in general lead to oxidative damage [1]–[3]. Increased oxygen exposure in 10- to 30-d saturation dives can also lead to increases in body iron stores [1], [3]–[6], and evidence exists that the status of vitamins—in particular, B vitamins involved in 1-carbon metabolism (for example, folate and vitamins B6 and B12)—could be affected by the oxidative environment [5]. In support of this concept, homocysteine was elevated during 10- to 12-d saturation dives [5]. Circulating homocysteine, a neurotoxin [7], increases in situations where folate, vitamin B12, or vitamin B6 status is decreased. An abundance of literature supports the notion that excess iron can also be toxic, through the formation of oxygen free radicals or increased availability of iron to pathogens or cancer cells, both of which are usually limited by iron availability [8]–[10].

Over the past 10 years, NASA has conducted NASA Extreme Environment Mission Operations (NEEMO) [3], [5] missions as an analog to simulate several aspects of spaceflight. In these 7-to 14-d missions, divers live in a habitat 19 m below the ocean surface in an atmosphere with an ambient pressure of 2.5 atm (253 kPa) and 21% oxygen. This hyperbaric, and thus oxygen-rich, environment associated with the NEEMO missions is ideal to identify and evaluate types of oxidative damage or repair in healthy individuals. In addition to oxidative damage, increases in iron stores, and changes in vitamin status [5], results from experiments on NEEMO missions have provided evidence for DNA damage (measured as an elevation in urinary 8-hydroxy 2′-deoxyguanosine) [3]. This is a concern for NASA because extravehicular activities (space walks) conducted from the International Space Station and proposed conditions for extravehicular activity on exploration missions in the future utilize environments with hyperoxic conditions.

In the study reported herein, we sought to characterize the impact of NEEMO missions on novel markers of oxidative damage to better identify the type of damage that occurs. Several markers of DNA damage and repair were measured, including serum high-mobility group box 1 (HMGB1) protein, a protein involved in an oxidative stress response and DNA repair [11]; γ-H2AX, a histone protein that is abundantly present in the repair response to DNA double-strand breaks [12], and poly(ADP-ribose) (PAR), which is formed in response to DNA damage [13].

In addition to documenting changes in DNA damage and repair responses, we also tested the hypothesis that folate status can change in as little as 13 d during a saturation dive, and that the changes are related to its antioxidant potential when body iron stores are elevated along with subsequent oxidative damage.

## Methods

The NEEMO XIV mission was a 13-d saturation dive that took place in May 2010. Four male divers from NASA and 2 male habitat technicians participated in the study. The habitat has been described in detail previously [3], [5].

The study was approved by the Johnson Space Center Committee for the Protection of Human Subjects. All participants were required to pass a modified Air Force Class III physical examination and were required to have logged a minimum of 25 dives before their selection as a crewmember. All subjects provided written informed consent.

Blood samples were collected 2 days before (designated pre-dive), during, and after the mission. Blood was collected on dive days 7 (mission day 7, denoted as MD7) and 13 (MD13) while the crew was in the underwater habitat. The blood tubes were placed on ice and transported to the surface, where a boat brought them to a nearby laboratory on shore for processing. The amount of time from the blood draw to the start of processing was 3–4 h. On splash-up day, blood samples were collected upon return of the crewmembers to shore (designated R+0), after they had undergone a 17-h decompression protocol.

Blood samples were analyzed for biomarkers of oxidative damage. Protein oxidation was assessed by utilizing liquid chromatography–tandem mass spectrometry (Kronos Science Laboratory, Phoenix, AZ) to measure 3-nitrotyrosine, which is formed by the modification of tyrosine residues during oxidative stress. Total lipid peroxides and malondialdehyde, well-established assays for screening and monitoring lipid peroxidation, were also measured as previously described [3]. To measure uremic toxins created during oxidative stress, advanced oxidation protein products were measured (ELISA kit, Cell Biolabs, Inc., San Diego, CA). As oxidative stress results from an imbalance in the levels of oxidants and antioxidants, the antioxidant defense enzymes superoxide dismutase (SOD), glutathione peroxidase (GPX), and catalase were measured to correlate their levels with the damage seen in the biomacromolecules, as previously described [3]. Catalase was determined by ELISA (Cayman Chemical, Ann Arbor, MI). Phosphorylated histone H2AX (γ-H2AX) and PAR were measured as previously described [12], [14]. HMGB1 was measured using a commercially available ELISA kit (IBL International GmbH, Hamburg, Germany). Total antioxidant capacity, glutathione, and selenium were measured to determine antioxidant status, as described previously [15]–[17].

Blood samples were also analyzed to perform a comprehensive evaluation of iron and hematological status. To test for the presence of hemolysis and an accompanying increase in iron storage, hemopexin, haptoglobin, bilirubin, heme, complete blood counts, hematocrit and hemoglobin, serum iron, ferritin, transferrin, transferrin receptors, labile plasma iron, and total iron-binding capacity were measured using standard clinical chemistry techniques, as described previously [5]. Body iron was estimated using an equation described by Cook et al. [18]. Serum hepcidin was measured using a commercially available kit (DRG Instruments GmbH, Germany). All samples had concentrations of hepcidin within the detection limits of the assay. Serum erythropoietin was measured using a chemiluminescent immunoassay (ARUP Laboratories, Salt Lake City, UT).

Blood samples were also evaluated for vitamin status. Specifically, RBC folate, serum folate, vitamin B12, homocysteine, cystathionine, 2-methylcitric acid, and methylmalonic acid (MMA) were measured to determine folate and vitamin B12 status using methods previously described [15]–[17].

Because of difficulties in aligning crew schedules, the post-dive sample collection, originally expected to occur 1 to 2 weeks after the dive, was not possible until 90 d after the dive (R+90). All samples were batch analyzed after this last collection, except for PAR and γ-H2AX, for which we had previously documented storage issues [12], [19] and which were analyzed within 2 weeks of splash-up.

The biochemical data were analyzed by repeated-measures analysis of variance with a post hoc Bonferroni test, to determine differences over time. Statistical analyses were performed using SigmaStat programs (Systat Software, Chicago, IL). Differences were considered significant at *P*<0.05.

## Results

Plasma catalase activity was greater during the dive than at baseline (*P*<0.05, Table 1), and at the end of the dive (MD13), whole-blood SOD was decreased from baseline (*P*<0.001, Table 1). At the end of the dive (R+0), plasma 3-nitrotyrosine was significantly lower than before the dive, but not different from after the dive (R+90). Whole-blood GPX did not change significantly.

**Table 1:** Whole blood and plasma variables related to oxidative damage, iron status, DNA damage, and vitamin metabolism before, during, and after a 13-d saturation dive in humans^1^.

Whole blood	Pre-dive	MD7	MD13	R+0	R+90
SOD^4^, *U/g hemoglobin*	1728±202^a^	1645±70^ac^	1218±190^b^	1280±242^b^	1313±115^bc^
GPX, *U/g hemoglobin*	50±11	50±10	49±11	51±11	59±9
White blood cell count, *10^9^/L*	6.1±0.7	6.9±1.1	6.5±0.9	6.8±1.1	5.9±1.1
RBC count, *10^12^/L*	4.7±0.2	4.8±0.3	4.3±0.9	4.7±0.3	4.8±0.1
Hematocrit, *%*	42.8±2.7	43.4±3.9	39.2±9.4	41.9±4.0	44.7±2.2
Hemoglobin, *g/L*	147±8	148±12	135±30	145±12	151±7
Platelet count, *10^9^/L*	203±46	209±37	201±71	200±36	197±27
PAR^3^, *pg/10^7^ cells*	81±8^a^	95±16^a^	122±21^ac^	176±70^bc^	
PAR^2^, *pg/µg actin*	21±4^a^	25±6^ac^	30±7^ac^	40±17^bc^	
**Plasma**					
Catalase^2^, *µmol ⋅ min^−1^⋅ L^−1^*	39±18^a^	51±29^ab^	71±10^b^	42±21^ab^	48±13^ab^
3-Nitrotyrosine^3^, *nmol/L*	0.2±0.1^a^	0.2±0.1^ab^	0.1±0.0^ab^	0.0±0.0^b^	0.1±0.0^ab^
Advanced oxidation protein products, *µmol/L*	301±74	375±125	303±61	311±111	201±92

1 Data are means ± SD, *n* = 6.

2,3,4 Significant effect of time, ^2^ *P*<0.05, ^3^ *P*<0.01, ^4^ *P*<0.001.

In each row, means without a common letter differ (*P*<.05) after a post hoc Bonferroni *t* test.

HMGB1 tended to decrease at the end of and after the dive (*P* = 0.06). PAR, expressed either as picograms per 10^7^ cells or per microgram actin, gradually increased during the dive and was significantly elevated immediately after the dive (Table 1). Gamma-H2AX was not detectable at any of the time points during or even before the dive (data not reported).

During the dive, white blood cell and RBC counts were not different from baseline, nor were hematocrit, hemoglobin, or platelet count (Table 1). RBC distribution width and mean corpuscular volume (MCV) decreased significantly during the dive (*P*<0.01 and *P*<0.001, respectively). Erythropoietin decreased early in the dive and stayed depressed throughout the dive (*P*<0.001, Table 2). Bilirubin and haptoglobin both tended to increase during the dive, but the changes were not significant. Serum iron was elevated during the dive (*P*<0.05).

**Table 2:** Serum variables related to oxidative damage, iron status, DNA damage, and vitamin metabolism before, during, and after a 13-d saturation dive in humans^1^.

	Pre-dive	MD7	MD13	R+0	R+90
Total antioxidant capacity, *mmol/L*	1.7±0.1	1.7±0.1	1.6±0.1	1.6±0.1	1.7±0
Total lipid peroxides, *µmol/L*	0.40±0.10	0.50±0.16	0.49±0.08	0.44±0.10	0.46±0.08
Erythropoietin^4^, *U/L*	6.8±1.7^a^	2.0±0.9^b^	3.5±1.9^b^	6.5±5.3^a^	6.3±1.9^a^
Iron^2^, *µmol/L*	21±6	22±9	25±4	25±3	16±3
Folate, *nmol/L*	39±8	40±8	38±7	44±20	42±9
Hepcidin^3^, *µg/L*	31.1±8.6^ab^	28.3±9.2^b^	27.9±8.6^b^	27.9±8.5^b^	33.5±9.4^a^
Total iron-binding capacity^2^, *µmol/L*	55±5^ab^	53±4^a^	50±4^b^	52±4^ab^	54±5^ab^
Transferrin saturation^3^, *%*	38±12^ab^	41±18^ab^	51±11^a^	48±7^a^	29±6^b^
Ferritin^4^, *pmol/L*	246±78^a^	369±103^b^	485±121^c^	494±127^c^	151±53^d^
Transferrin^4^, *g/L*	2.45±0.19^a^	2.44±0.21^a^	2.25±0.17^b^	2.32±0.21^a^	2.45±0.25^a^
Transferrin receptors^3^, *mg/L*	4.0±0.8^a^	4.0±0.7^a^	3.3±0.6^b^	3.7±1.0^a^	4.5±1.0^a^
Estimated body iron^4^, *mg/kg*	10±2^a^	12±1^b^	14±1^c^	13±1^c^	8±2^d^
Selenium, *µmol/L*	2.21±0.12	2.21±0.15	2.11±0.15	2.27±0.16	2.11±0.14
HMGB1, *µg/L*	22±4	25±10	18±5	16±2	17±3
Bilirubin, *µmol/L*	7±2	8±1	7±2	6±2	7±2
Haptoglobin, *g/L*	1.01±0.66	1.19±0.57	1.22±0.50	1.26±0.48	0.88±0.59
2-Methylcitric acid^2^, *nmol/L*	158±41^a^	182±49^ab^	200±22^b^	184±39^ab^	167±34^ab^
MMA^2^, *nmol/L*	145±20^ab^	146±23^ab^	138±24^a^	168±15^b^	162±22^ab^
Cystathionine, *nmol/L*	124±37	112±34	124±26	144±31	124±43
Homocysteine, *µmol/L*	7.5±1.0	8.2±1.6	7.4±0.9	7.3±1.1	7.1±2.0
MCH, *pg/cell*	31.1±1.0	30.8±0.8	31.1±0.6	30.8±0.7	31.3±0.7
MCHC, *g/L*	344±3	342±3	347±9	346±5	338±3
MCV^4^, *fL*	90.4±3.3^ac^	90.2±3.0^ac^	89.6±3.8^ab^	88.9±3.4^b^	92.5±2.9^c^
RBC folate^4^, *nmol/L*	1466±331^a^	1289±284^ac^	1039±291^b^	1116±291^bc^	1422±218^ac^
Vitamin B12^4^, *pmol/L*	424±72^a^	494±64^b^	489±82^b^	484±90^b^	452±52^c^

1 Data are means ± SD, *n* = 6.

2,3,4 Significant effect of time, ^2^ *P*<0.05, ^3^ *P*<0.01, ^4^ *P*<0.001.

In each row, means without a common letter differ (*P*<.05) after a post hoc Bonferroni *t* test.

Total iron-binding capacity and transferrin were both lower at the end of the dive than at the beginning (Table 2). Transferrin saturation was higher during the dive than at the post-mission time point (R+90). The circulating concentration of transferrin receptors was lower during the dive (*P*<0.01) and ferritin was higher during the dive, with its highest concentration at the end of the dive (*P*<0.001). Estimated body iron stores increased during the dive as well (*P*<0.001). Hepcidin, a regulator of iron absorption in the small intestine and iron release from macrophages, was lower during the dive than at R+90 (*P*<0.01).

The concentration of folate in RBC had decreased at the end of the dive (*P*<0.001), but serum folate was not significantly different from baseline. Vitamin B12 was elevated during the dive and lower 3 months after the dive, but the concentration was still greater than at baseline. Cystathionine and homocysteine were not different during the dive, but 2-methylcitric acid was elevated during the dive compared to the predive concentration (*P*<0.05). RBC folate was inversely correlated with 2-methylcitric acid (Pearson *r* = −0.77, *P*<0.001) and with serum ferritin (Pearson *r* = −0.34, *P*<0.05). Serum vitamin B12 was not related to 2-methylcitric acid or MMA.

## Discussion

We have expanded our previous work [5] characterizing the effect of a saturation diving environment on oxidative damage and iron and folate metabolism. Moreover, this study documents evidence of DNA damage repair, given the steady increase in PAR over the course of the dive, suggesting that DNA damage occurred during the dive at some point. HMGB1 tended to decrease (*P* = 0.06) during the dive, which provides supporting evidence for a DNA repair response. H2AX is a histone protein that becomes rapidly phosphorylated on a serine 4 residues from the carboxyl terminus (serine c-4) to form γ-H2AX at nascent double-strand DNA break sites [20], [21]. Immediately after double-strand break formation, there is a large increase in the number of γ-H2AX molecules in the chromatin around the break site. The fact that γ-H2AX was not detectable at any of the time points suggests that the DNA damage did not involve double-strand breaks. There might not have been enough DNA break accumulation in the peripheral blood mononuclear cells, which are relatively nonproliferative in the absence of mitogen stimulation. Because most double-strand DNA breaks occur at the replication fork, the lack of proliferation of this cell type may be a potential reason that these types of breaks (and repair processes) were not observed in this type of sample. Other considerations could be the timing of the sample collection or signal preservation during sample processing (due to the ∼4 h delay in processing after sample collection, as mentioned in the Methods section). Nonetheless, the data presented here suggest that PAR may be a sensitive biomarker indicating the presence of an oxidative insult.

With regard to iron and hematological adaptation, the NEEMO environment has proven a reliable and consistent analog for the body iron changes observed during spaceflight and descent from altitude [22], [23]. These environments trigger an increase in iron stores. An initiating event in this process seems to be the reduction in erythropoietin [24], which was observed here during the dive. Within hours of return to a nominal pressure and oxygen environment, this hormone returned to predive concentrations. Suppression of erythropoietin has been documented in descent from altitude [22] and during spaceflight [23]. Neocytolysis and excess iron availability are also hallmarks of these physiological stressors [24]. In the present study, serum iron increased during the dive, as did serum ferritin. Similar to our results here, Rice et al. [22] found that haptoglobin did not change upon descent from high altitude, but there was evidence for increased body iron stores, decreased erythropoietin, and decreased RBC mass. We did not measure RBC mass, but the decrease in MCV during the dive provides evidence for a decrease in the number of newly formed RBC, which are larger.

Because RBC survive for an average of 120 d, RBC folate is considered a stable, long-term indicator of folate status, and unlike serum folate does not reflect recent intake. Therefore, the rapid decrease in RBC folate observed here is likely due to factors other than possible dietary changes. In a previous study [5], the amount of variability in the RBC folate data (the crew consisted of 4 men and 2 women) was considerable, and although folate status tended to decrease during the dive, the change was not significant. We hypothesized that the decrease would reach significance with a larger sample size. In the present study, a consistent decrease in RBC folate of all crewmembers was significant. One explanation of this finding may be that folate acts as an antioxidant in this oxidative environment and therefore the requirement for it is increased. Several *in vitro* studies have shown that the reduced form of folate can act as an antioxidant [25]–[28]. Another possible explanation is that the increased concentration of ferritin acted directly to catalyze folate turnover. *In vitro* studies provide evidence that the heavy chain of ferritin can catabolize folate [29]. Supporting this is the finding, in the current study, that folate status was negatively correlated with ferritin (*P*<0.05) and positively correlated with transferrin receptors (*P*<0.01). Interestingly, and parallel with the NEEMO findings, ferritin increases during and after spaceflight, and folate status decreases after long-duration spaceflight [30].

We expected an increase in homocysteine to occur during the dive, similar to what was observed during a previous NEEMO mission [5], but no increase was observed here. The lack of difference between values of some of the outcome variables measured in this study and their values in the previous NEEMO mission may be attributable to a smaller sample size and different operational mission objectives (some NEEMO missions may have more or longer dives scheduled, either in the pre-dive training phase or during the dive itself). During the dive, 2-methylcitric acid, a downstream metabolite in the homocysteine transsulfuration pathway [31], was elevated, and it is usually elevated during a vitamin B12 deficiency. A vitamin B12 deficiency was not observed in this study, but a difference in dietary patterns or an unknown environmental factor might have pushed this pathway toward transsulfuration instead of remethylation. Additional data from another experiment would be needed to explain the lack of increase in homocysteine.

This study provides evidence that folate requirements may be greater for individuals with sustained increases in ferritin. It also shows that many markers of oxidative damage were already normalized by the end of the dive, or perhaps are not stable or sensitive enough to be used as reliable markers. Some markers, however, remained changed from baseline after 13 d of diving, and could be used as biomarkers to monitor and test countermeasures for mitigating oxidative damage. NEEMO provides an excellent model to study changes in iron metabolism and oxidative damage resulting from it. NEEMO missions of longer duration need to be conducted to know if these markers of oxidative damage eventually normalize and whether the changes in iron and folate status continue or plateau. Knowing the answers to these questions is critical for future exploration space missions, where crews may be exposed to long or frequent periods of hyperoxic conditions.
